# Power and precision of QTL mapping in simulated multiple porcine F2 crosses using whole-genome sequence information

**DOI:** 10.1186/s12863-018-0604-0

**Published:** 2018-04-03

**Authors:** Markus Schmid, Robin Wellmann, Jörn Bennewitz

**Affiliations:** Institute of Animal Science, University of Hohenheim, 70599 Stuttgart, Germany

**Keywords:** Genome-wide association studies, Mapping power, Mapping precision, Pooling data, Porcine F2 crosses, Simulation study, Whole-genome sequence data

## Abstract

**Background:**

During the last two decades, many QTL (quantitative trait locus) mapping experiments in pigs have been conducted using F2 crosses established from two outbred founder breeds. The founder breeds were frequently chosen from the Asian and European type breeds. A combination of next-generation sequencing, SNP (single nucleotide polymorphism) genotyping technology using SNP-chips, and genotype imputation techniques, can be used to infer the sequence information of all F2 individuals in a cost-effective way. The aim of the present simulation study was to analyze the power and precision of genome-wide association studies (GWASs) with whole-genome sequence data in several types of F2 crosses, including pooled crosses.

**Methods:**

Based on a common historical population, three breeds representing two European type breeds (EU1 and EU2) and one Asian type breed (AS) were simulated. Two F2 designs of 500 individuals each were simulated. The cross EU1xEU2 (ASxEU2) was simulated using the phylogenetically closely related breeds EU1 and EU2 (or distantly related breeds AS and EU2) as the founder breeds. The simulated genomes comprised ten chromosomes, each with a length of 1 Morgan and whole-genome sequence information. A polygenic trait with a heritability of 0.5, which was affected by approximately 20 QTL per Morgan, was simulated. GWASs were conducted using single marker mixed linear models, either within the crosses or in their pooled datasets. Additionally, the studies were conducted in the breed EU2, which was a founder breed in both simulated crosses.

**Results:**

The power to map QTL was high (low) in the ASxEU2 (EU1xEU2) cross and was highest when the data of both crosses were analyzed jointly. By contrast, the mapping precision was the highest in the EU1xEU2 cross. Pooling data led to a precision that was in between the precision of the EU1xEU2 cross and the ASxEU2 cross. A higher mapping precision was observed for QTL segregating within a founder breed.

**Conclusions:**

These results suggest that the existing F2 crosses are promising databases for QTL mapping when the founder breeds are closely related or several crosses can be pooled. This conclusion is particularly applicable for QTL that segregate in a founder breed.

## Background

QTL (quantitative trait locus) mapping and the identification of causative single nucleotide polymorphisms (QTNs, quantitative trait nucleotides) is still of high importance in animal breeding. The results of genome-wide association studies (GWASs) provide knowledge about the evolution and genetic architecture of traits and may improve the accuracy of genomic prediction [1], especially if the studies rely on genomic sequence data [2]. Before large-scale single nucleotide polymorphism (SNP) genotyping using next-generation sequencing technology was possible in pig breeding, QTL mapping was frequently performed by applying linkage analyses using sparse genetic maps, which were often built by microsatellite markers. The necessary linkage disequilibrium (LD) was established within families by generating experimental crosses. Many pig F2 crosses have been generated during the last few decades, and numerous QTL for various traits have been reported [3, 4]. Often, the F2 individuals were phenotyped under standardized conditions (e.g., on experimental farms) for interesting but hard-to-measure traits, such as efficiency traits or meat quality traits. Founder breeds were frequently chosen from Asian and European pig breeds. Phylogenetic analysis of whole-genome sequence data revealed distinct lineages of these two types of breeds [5]. However, F2 crosses within European breeds were also established (e.g., [6]). In many cases, a commercially used breed was one of the two founder breeds. For example, the F2 crosses described in [6, 7] both had Piétrain as one founder breed, which is an important sire line breed in Europe.

Since the availability of dense SNP maps and the possibility to conduct large-scale SNP genotyping with SNP-chips, QTL mapping is usually performed in genome-wide association studies (GWASs) within breeding populations or in admixed populations [1]. For example, QTL mapping was performed in the Piétrain breed (mentioned above) by Stratz et al. [8] using the Illumina PorcineSNP60 Beadchip [9]. Ledur et al. examined whether it is worthwhile to conduct large-scale SNP genotyping in F2 crosses [10]. They studied the power of GWAS in F2 crosses that were genotyped with large-scale SNP maps using simulations and compared the results with classical linkage analysis mapping. Their findings showed an increase in power and a smaller rate of false positive results in F2 crosses with large sample sizes and high marker densities. A recent simulation study analyzed the mapping resolution and the linkage disequilibrium structures around causal genes of several simulated pig F2 crosses at a maximized marker density (sequence information available for all individuals) [11]. It was shown that the mapping resolution is high for genes that are also segregating in a founder breed, especially for F2 crosses established from two closely related founder breeds. In a few cases, the mapping resolution was even higher compared with a single outbred founder population due to the variation of LD between markers and QTNs among the founder breeds. Toosi et al. [12] reported similar results from a simulation of admixed cattle genomes. Thus the numerous past established F2 crosses might be underused experimental populations for mapping QTL and QTNs. This hypothesis might especially hold true for QTNs that segregate in the founder breeds. These QTNs are of interest for improving genomic predictions conducted within the founder breed. For example, mapping Piétrain segregating QTNs could improve the accuracy of genomic selection, which was implemented in this breed [13].

The aim of the present study was to analyze the power and precision of GWASs with whole-genome sequence data in several types of F2 crosses, including pooled crosses. Particular emphasis was paid to founder breed segregating QTNs because these are of interest for breeding purposes. The crosses were established using distantly or closely related founder breeds using stochastic simulations. The results were compared with those obtained from pooled F2 crosses, which increased the sample size and putatively reduced the LD. For comparison purposes, we also simulated one of the founder breeds and conducted GWASs within this breed.

## Methods

### Simulation of founder and F2 cross individuals

**Fig. 1:**
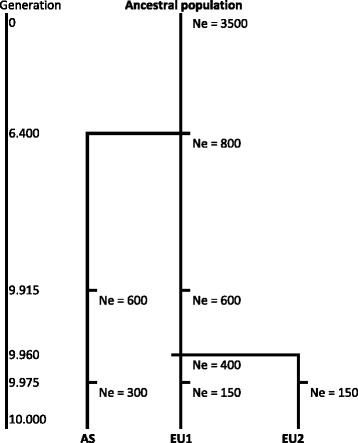
Phylogenetic history of the simulated founder breeds AS, EU1, and EU2

Two porcine F2 crosses were simulated, one with closely related founder breeds and the other with distantly related founder breeds. One founder breed was the same in both crosses. A forward simulation approach was used to generate a Fisher-Wright diploid ancestral population, from which the founder breeds descended. The protocol to simulate the founder breeds is based on the knowledge of the phylogeny of pig breeds, especially the distinct lineages of European breeds and Asian breeds [5] and a sharp reduction of the effective population size over time due to intensified breeding schemes [14]. This protocol is described in detail in the following section and is also shown in Fig. 1. The ancestral population was simulated for 6400 generations with an effective population size (Ne) of 3500. In this generation, the ancestral population was split into two distinct lineages: the European and Asian lineages. These lineages were simulated independently from each other from this generation onward. The Asian lineage was simulated until generation 9915 with a Ne of 800, from generation 9915 until generation 9960 with a Ne of 600 and 9975 until 10,000 generations with a Ne of 300. The last generation represented the Asian founder breed (AS). The European lineage was simulated from generation 6400 until generation 9915 with a Ne of 800 and from generation 9915 until generation 9960 with a Ne of 600. In this generation, two breeds were generated from this lineage (breeds EU1 and EU2), which were simulated independently, until generation 9975 with a Ne of 400 and from generation 9975 until generation 10,000 with a Ne of 150. The level of genetic differentiation of the founder breeds was assessed by estimating the population differentiation index F_ST_, using the formula (8) in Weir and Cockerham [15].

**Fig. 2:**
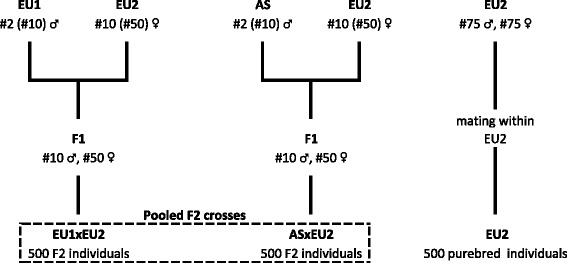
F2 schemes. F2 schemes derived from phylogenetically closely (left) and distantly (middle) related founder breeds based on a small (large) number of F0 individuals as well as two generations of mating EU2 (right) as the purebred experimental population

From the three simulated founder breeds, two F2 crosses were generated, one with the closely related founder breeds EU1 and EU2 (EU1xEU2) and the other one with the distantly related founder breeds AS and EU2 (ASxEU2). The EU1xEU2 cross was established as follows. Founder animals were randomly selected from the founder breeds. The number of founder animals to establish an F2 crosses varied in real experiments, with usually a lower number of males compared to females (e.g. [6, 7]). In order to mimic this variable number of founder animals in our simulation, two different numbers were selected: two and ten males were selected from EU1 and ten and 50 females were selected from EU2. These animals were mated to create ten male and 50 female F1 offspring. Each F1 male was mated to five F1 females with an assumed litter size of ten. Each female was allocated to only one male. This mating scheme resulted in 500 F2 EU1xEU2 individuals. Hence, we simulated two EU1xEU2 crosses, one with many and one with few founder animals. The same protocol was used to simulate the ASxEU2 cross; however, AS was the paternal founder breed. Both crosses shared one founder breed (EU2), but the founder animals from this breed were different. The datasets were pooled for the joint analyses of both crosses, also shown in Fig. 2.

### Genomes and traits

Ten chromosomes of one Morgan (M) length each were simulated. The pig genome consists of more than ten chromosomes, but we restricted this number for computational reasons. Recombination events were simulated according to the Haldane mapping function. The mutation rate was adjusted in a simplified manner so that two mutations per chromosome (20 per genome), on average, were expected to occur per meiosis. All SNPs were generated solely by the mutations within the evolution of the simulated populations. This protocol was repeated ten times. For each population, five traits were simulated, which resulted in 50 replicates in total. For each trait, 20 SNPs per chromosome were randomly selected to become a QTN, which resulted in 200 QTN to mimic the polygenic nature of quantitative traits [13]. Because the QTN were randomly selected, the traits were assumed to be unselected. This might be a simplification, because in reality some traits in F2 crosses are under selection in the founder breeds. However, considering this in a simulation is not straightforward and would result in additional assumptions. The minimum distance between QTNs was 2 Centimorgan (cM). The additive effects were sampled from a *t*-distribution with four degrees of freedom and were assumed to be the same for the two single crosses. This result roughly resembled the distribution of additive effects in porcine F2 crosses [16]. Breeding values were calculated for the individuals as follows [17]. For an individual with genotype x (x representing the number of copies of the mutant allele at QTN, x = 0, 1, or 2), the breeding value (BV) is

$$ BV(x)=\sum \limits_{j=1}^Q\left({x}_j-2{p}_j\right){a}_j, $$

with *a*_*j*_ being the simulated additive effect, *p*_*j*_ the frequency of the mutant allele, and Q the number of simulated QTN. The additive genetic variance was calculated as the variances of the breeding values within the pooled dataset. Hence, the additive genetic variance differed slightly between the crosses due to different gene frequencies at the QTNs. The gene frequencies were more intermediate in the ASxEU2 cross compared to the EU1xEU2 cross, because in the former cross the two founder breeds were less related. However, in general the difference of the gene frequencies in both crosses were small. A random residual was added to the breeding values to complete the phenotypes of the individuals, assuming a heritability of 0.5 in the pooled dataset. In addition, 1000 EU2 individuals were simulated using the same procedures. Note that the LD structure of these types of simulated F2 crosses were investigated in detail in an earlier study [11], and hence it was not included in this study.

### Association mapping

All SNPs and QTNs with a minor allele frequency (MAF) below 0.05 in the individual crosses were removed from the following analyses. GWASs were conducted for each SNP (also for each QTN) separately by using the following regression model and the software GCTA (Genome-wide Complex Trait Analysis) [18]:

$$ {y}_i={\mu}_{k_i}+{b}_j{x}_{ij}+{g}_i+{e}_i. $$

Here, *y*_*i*_ denotes the phenotypic value of individual *i*, ${\mu}_{k_i}$ denotes the overall mean of the cross *k* to which individual *i* belongs, *x*_*ij*_ denotes the number of copies of a randomly chosen allele of SNP *j* (*x*_*ij*_ = 0, 1, or 2) and *b*_*j*_is the regression coefficient for SNP *j*. The random polygenic effect of the individual (*g*_*i*_) was fit to capture population stratification effects. The covariance structure of the polygenic effects was modeled using a genomic relationship matrix (GRM) [18]. To avoid the pitfall of double fitting the SNP to be tested simultaneously as a fix and a random effect, a leave-one-chromosome-out approach was applied, as recommended [18]. This approach meant that when the SNP effects were tested for significance on a certain chromosome, the SNPs on this chromosome were excluded from the calculation of the GRM. The correction for multiple testing was conducted using the Bonferroni method. The two crosses were analyzed both separately and jointly (i.e., the pooled datasets). The slightly larger additive genetic variance of the cross ASxEU2 was accommodated by the GRM, in which the off-diagonal elements were larger for the individuals in this cross compared with the corresponding elements of the EU1xEU2 cross.

### Scenarios

Two GWAS scenarios were considered. In the *all segregating genes* (ASG) scenario, the aim was to map all available QTNs. Association mapping was performed on the full set of SNPs and QTNs. However, from a breeder’s perspective, GWAS results are most important for QTNs segregating in the breed of interest. As economically relevant breeds (e.g., the Piétrain breed) were often used as founder breeds in F2 crosses, a second scenario, the so-called *founder segregating genes* (FSG) scenario was considered. The aim was to map QTNs that segregated in the common founder breed EU2. Consequently, all SNPs and QTNs that did not segregate in the common founder breed EU2 were removed from the simulated datasets because they could be excluded beforehand as putative QTNs. The association analyses were subsequently conducted using these reduced data sets. Consequently, the number of tests were much smaller and so were the levels of multiple testing corrections using Bonferroni. Both GWAS scenarios (ASG and FSG) were applied to all simulated F2 crosses.

For comparison purposes, the simulated purebred EU2 data set was also analyzed.

### Calculation of QTN and QTL mapping power and QTN mapping precision

The set of SNPs was denoted as *S*. In the ASG scenario, *S* contained all segregating SNPs (MAF > 0.05 in the respective cross), but in the FSG scenario, the set included only SNPs also segregating in the common founder breed (MAF > 0.05 in EU2). Thus, *Q*_*α*_ ⊆ *Q* ⊆ *S*, where *Q* is the set of QTNs, and *Q*_*α*_ contains all simulated QTNs with a Bonferroni corrected *p*-value less than *α*. We calculated the power to map QTNs as the proportion of QTNs with a Bonferroni corrected *p*-value smaller than *α*, i.e., $QTN\  power=\frac{\#{Q}_{\alpha }}{\#Q}$, where #*Q* denotes the number of elements in set *Q*. This definition is in agreement with classical statistical test theory. QTL power was defined as the proportion of QTNs, which are either mapped per se or by a significant SNP in LD with the QTN To determine whether a QTN *i* can be detected through a SNP in high LD, a window *W*_*i*_ was defined spanning 1 cM with the QTN in the center. This window defined the QTL region. If the SNP with the smallest *p*-value was significant in such a QTL window, the QTN was indicated by this SNP and, therefore, was mapped. Hence, the QTL power was calculated as $QTL\  power=\frac{\#{W}_{\alpha }\ }{\#Q}$, with #*W*_*α*_ being the number of windows, which contained a QTN and at least one significant SNP within these windows.

The windows were also considered to specify the precision of mapping QTNs. For each window containing a significant QTN, the proportion of SNPs showing a higher significance than the QTN itself was computed. The QTN mapping precision was then calculated by subtracting this proportion from 1. This step ensured that the maximum achievable mapping precision was one, which implied that the QTN showed the highest significance among all SNPs in the window. By contrast, a precision close to 0.5 indicated that 50% of the significant SNPs were more significant than the causal mutation.

The parameters QTN power, QTL power, and QTN precision were calculated for each analyzed data set and then averaged across the simulated replicates.

## Results and discussion

### Simulation structure

Maximum marker density was simulated, which resembles a situation where the whole genome sequence variants are known from each F2 individual. In real porcine F2 crosses, sequencing all F2 individuals is still unaffordable, but the Illumina PorcineSNP60 BeadChip [9] with approximately 62 k SNPs can be used to impute sequence data from founder individuals in the F1 and subsequently in the F2 generation utilizing mainly pedigree information. Thus, the sequence data of F2 individuals can be generated by sequencing the founder individuals and SNP-chip genotyping the F1 and F2 generation, which is affordable in many situations. Although this strategy was not evaluated so far, it can reasonably be assumed that the imputation accuracy will be high.

The F_ST_ value calculated between the two simulated European breeds EU1 and EU2 was F_ST_ = 0.02, and between the Asian breed AS and the European breed EU2 it was F_ST_ = 0.36. These values implied a small (large) genetic differentiation between EU1 and EU2 (AS and EU2) [19]. Hence, although simplified assumptions during the establishment of the simulation protocol had to be made, it fits roughly the genetic differentiation of typical real pig founder breeds.

**Fig. 3:**
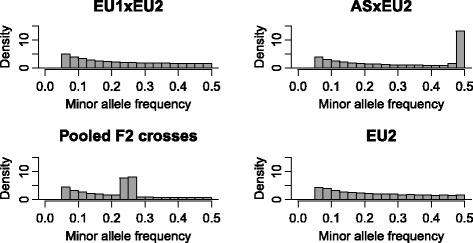
Minor allele frequencies for a randomly chosen trait. Minor allele frequencies for all SNPs with a MAF > 0.05 within the two F2 crosses (top line), their pooled data and the purebred experimental population (bottom line)

The average number of SNPs across all replicates in the ASG scenario with an MAF > 0.05 within the respective populations is given in Table 1. The MAF of SNPs with a MAF > 0.05 within the experimental populations are shown in Fig. 3 for a randomly chosen replicate. In most scenarios, the number of segregating SNPs in the F2 designs was higher compared with the founder population even though the numbers of founder individuals of the F2 crosses were limited. This increase was substantial, especially in the ASxEU2 cross. This was due to the numerous SNPs that were divergently fixed (or close to fixation) in the distantly related founder breeds but was segregating in the F2 cross, as shown in Fig. 3. Pooling data from both designs increased the number of SNPs only slightly (Table 1). The LD structure of the simulate crosses can be found in [11].

**Table 1:** Number of SNPs (MAF > 0.05) within the respective datasets for the ASG and FSG scenarios

Scenario	ASG		FSG	
	mean	sd	mean	sd
EU2	100,783	683		
EU1xEU2 (small F0)	97,490	974	83,228	717
EU1xEU2 (large F0)	104,797	965	89,192	657
ASxEU2 (small F0)	237,574	698	79,106	619
ASxEU2 (large F0)	247,726	1026	81,688	657
Pooled F2 crosses (small F0)	248,302	1805	86,444	693
Pooled F2 crosses (large F0)	240,115	897	89,607	746

The means and standard deviations across all simulated datasets are shown

For the FSG scenario, the average numbers of SNPs with an MAF > 0.05 in the EU2 and the F2 crosses are given in Table 1. The numbers of SNPs were similar in all crosses and were lower than the number for the purebred population. The smallest number was observed in ASxEU2, which was derived from a small number of F0 individuals, because AS had many private alleles, and, therefore, shared fewer SNPs with the EU2 breed. A higher number of SNPs could be observed if the F2 designs were based on a larger number of founder individuals. The number of SNPs was the highest in pooled data.

### Mapping power and precision

The power to detect a QTN or at least one significant SNP within a 1 cM window around a QTN (i.e., a QTL) is given in Table 2 for the ASG scenario. This result showed that the mapping power was higher in ASxEU2 than in EU1xEU2. That was attributed to various mutations that were divergently fixed in the distantly related founder breeds and therefore were segregating with a high MAF in the F2 generation. By contrast, QTNs segregating in EU1xEU2 had more extreme allele frequencies. Because the QTL contributions to the total genetic variance strongly depended on allele frequencies, the power in ASxEU2 was substantially higher than in EU1xEU2. Additionally, the LD blocks are larger F2 crosses derived from distantly related founder breeds (like the ASxEU2) [11], and several QTNs may have been in LD with the QTN being tested, which may increase the effect explained by the QTN. Hence, the mapping power (especially QTL mapping power) is higher in such designs where more SNPs are in LD with a QTN. The power was highest when the datasets were pooled and analyzed jointly, which resulted from the larger sample size. The mapping power depended only slightly on the number of founder individuals (Table 2).

**Table 2:** QTN and QTL mapping power and QTN mapping precision in the ASG scenario at a genome-wide significance level of *α* = 0.05

Parameter	QTN Power		QTL Power		QTN Precision	
	mean	sd	mean	sd	mean	sd
EU1xEU2 (small F0)	0.014	0.017	0.019	0.027	0.784	0.183
EU1xEU2 (large F0)	0.009	0.012	0.012	0.014	0.823	0.098
ASxEU2 (small F0)	0.064	0.044	0.127	0.081	0.672	0.105
ASxEU2 (large F0)	0.063	0.040	0.122	0.068	0.619	0.116
Pooled F2 crosses (small F0)	0.070	0.047	0.141	0.088	0.678	0.108
Pooled F2 crosses (large F0)	0.070	0.042	0.134	0.074	0.661	0.134

The means and standard deviations across all simulated replicates are shown

A low mapping precision was observed in F2 crosses with phylogenetically strongly divergent founder breeds (Table 2). This is because the number of divergently fixed alleles was very high (Fig. 3) and, therefore, LD blocks large. The pooling of data resulted in a precision that was between the precision of both F2 crosses.

**Fig. 4:**
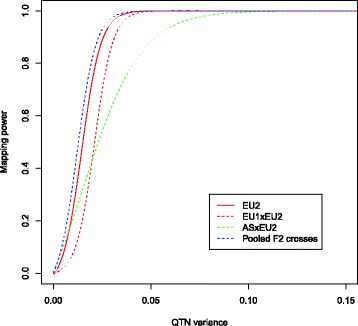
QTN mapping power as a function of the QTN variance. Mapping power as a function of the QTN variance (contribution to the phenotypic variance (VP) averaged across all replicates

In the FSG scenario, the EU2 was the breed of interest, and the aim was to map QTNs segregating in this breed. The results for QTL and QTN power in this scenario are shown in Table 3. In ASxEU2, the QTN power was higher than in EU1xEU2. The reason were the same as in the ASG scenario, such as more QTNs may have intermediate allele frequencies and several QTNs may be in LD with the QTN being tested. Pooling the data again led to an increase in power due to a larger sample size. However, it could not reach the mapping power in the purebred population at an equal sample size whose trait was simulated to have the same heritability. The reason for this result may be that the distribution of allele frequencies was U-shaped in the purebred population. Consequently, the distribution of the contributions of QTNs to the phenotypic variance was more heavy-tailed, as it can be seen in Fig. 3. The QTN mapping power as a function of the QTN size is shown in Fig. 4. The QTL power was substantially larger than the QTN power in crosses with distantly related founder breeds and a small number of founders because more SNPs were in strong LD with the QTNs.

**Table 3:** QTN and QTL mapping power and QTL mapping precision in the FSG scenario at a genome-wide significance level of *α* = 0.05

Parameter	QTN Power		QTL Power		QTN Precision	
	mean	sd	mean	sd	mean	sd
EU2 (500)	0.031	0.015	0.037	0.017	0.748	0.202
EU2 (1000)	0.076	0.026	0.090	0.028	0.874	0.080
EU1xEU2 (small F0)	0.015	0.019	0.020	0.026	0.758	0.191
EU1xEU2 (large F0)	0.011	0.014	0.013	0.016	0.834	0.093
ASxEU2 (small F0)	0.036	0.031	0.125	0.081	0.725	0.212
ASxEU2 (large F0)	0.033	0.024	0.114	0.068	0.626	0.195
Pooled F2 crosses (small F0)	0.050	0.036	0.138	0.080	0.788	0.181
Pooled F2 crosses (large F0)	0.038	0.024	0.113	0.065	0.757	0.182

The means and standard deviations across all simulated replicates are shown

As shown in Table 3, the precision in the FSG scenario was the highest for the EU1xEU2 cross with a large number of founder animals. The precision was even higher than in the purebred EU2 population with 500 individuals in the analysis. The high precision of the closely related cross resulted from the fact that LD blocks in crossbred populations may have been shorter than in the purebred populations [12]. The lowest precision was observed in the crosses of distantly related breeds.

The precision in the FSG scenario was always above the precision in the ASG scenario. This result is in agreement with [11] for which the highest mapping resolution was observed in F2 populations for genes that also segregated in a founder breed.

**Fig. 5:**
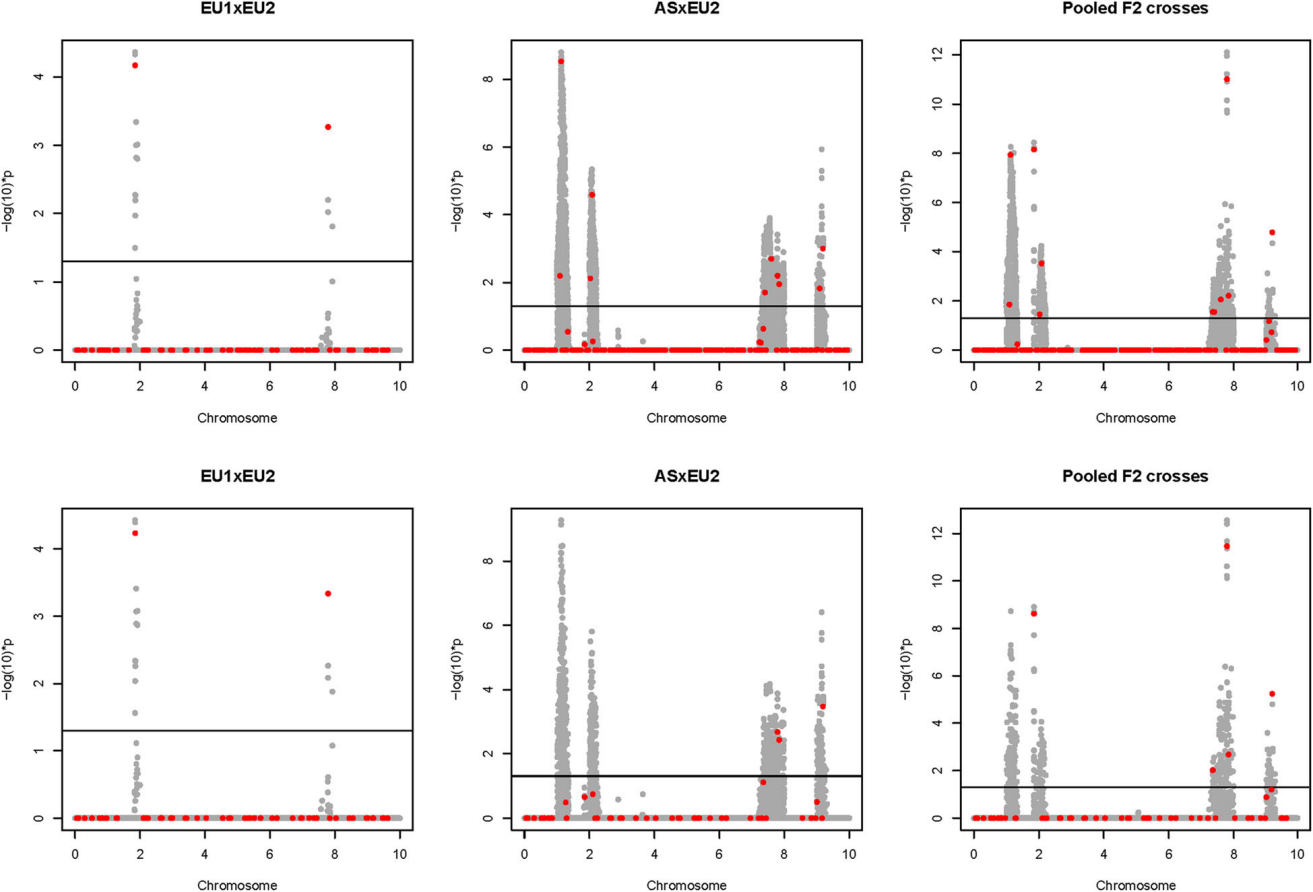
Manhattan plots of both scenarios for a randomly chosen replicate. Test statistics (−log (10)*p-value) and the position of SNPs (gray dots) and QTN (red dots) segregating in the F2 crosses (ASG scenario, top line) and also within the common founder breed EU2 (FSG scenario, bottom line) for both F2 crosses and their pooled data. The solid line corresponds to a genome-wide significance level of *α* = 0.05

The general pattern of the mapping power and precision results in the simulated populations and scenarios as described above is visualized by a comparison of the Manhattan plots for a randomly chosen replicate in Fig. 5.

## Conclusions

Based on the results of this simulation study, in can be concluded, that the existing F2 crosses are promising databases for gene mapping in the era of genomics when the founder breeds are closely related or when crosses can be pooled. For the fine-mapping of QTNs, F2 crosses from distantly related founder breeds should be pooled with data from additional populations in which the QTNs of interest are segregating. This step could substantially increase the precision. By contrast, the mapping precision could be even higher in F2 crosses from closely related founder breeds than in the purebred population; thus, no pooling would be required if the sample size and the numbers of founders in the F0 are sufficiently high. This conclusion is particularly true for QTNs that segregate in a founder breed.

## Abbreviations

AS: Asian founder breed
ASG: All segregating genes
ASxEU2: F2 cross derived from distantly related founder breeds
BV: Breeding value
cM: Centimorgan
EU1: European founder breed 1
EU1xEU2: F2 cross derived from closely related founder breeds
EU2: European founder breed 2
FSG: Founder segregating genes
F_ST_: Population differentiation index
GCTA: Genome-wide complex trait analysis
GRM: Genomic relationship matrix
GWAS: Genome-wide association study
LD: Linkage disequilibrium
M: Morgan
MAF: Minor allele frequency
Ne: Effective population size
QTL: Quantitative trait locus
QTN: Quantitative trait nucleotide
SNP: Single nucleotide polymorphism
